# Evaluating the validity of a functional electrical stimulation clinical decision making tool: A qualitative study

**DOI:** 10.3389/fneur.2022.1001123

**Published:** 2022-11-15

**Authors:** Nathalie Abouzakhm, Samantha Choy, Rebecca Feld, Chris Taylor, Kathryn Carter, Spencer Degroot, Kristin E. Musselman

**Affiliations:** ^1^Department of Physical Therapy, Temerty Faculty of Medicine, University of Toronto, Toronto, ON, Canada; ^2^KITE, Toronto Rehabilitation Institute-University Health Network, Toronto, ON, Canada; ^3^Rehabilitation Sciences Institute, Temerty Faculty of Medicine, University of Toronto, Toronto, ON, Canada

**Keywords:** physical therapist, occupational therapist, functional electrical stimulation, qualitative research, evidence-based practice, neurorehabilitation

## Abstract

**Introduction:**

Following central nervous system damage, the recovery of motor function is a priority. For some neurological populations, functional electrical stimulation (FES) is recommended in best practice guidelines for neurorehabilitation. However, limited resources exist to guide FES application, despite clinicians reporting that a lack of FES knowledge prevents use in clinical practice. The FES Clinical Decision Making Tool was developed to assist clinicians with FES application and translation into clinical practice. The purpose of this study was to evaluate the content validity of the Tool from the perspectives of Canadian physical and occupational therapists using FES in neurorehabilitation.

**Methods:**

Thirteen participants (twelve women, one man), aged 40.5 ± 10.3 years, participated in individual semi-structured interviews to explore their clinical decision making experiences when applying FES and to evaluate the content validity (i.e., appropriateness, comprehensibility, and comprehensiveness) of the Tool. Interviews were analyzed using a qualitative conventional content analysis following the DEPICT model.

**Results:**

Three themes were identified. 1) Clinician context influences FES usage. Participants' experiences with FES use varied and application was influenced by treatment goals. 2) Parameter selection in clinical practice. Participants identified decision-making strategies and the challenges of parameter selection. 3) With modifications, the Tool is a valid resource to inform FES applications. Participants discussed its strengths, limitations, and suggested changes. While the Tool is useful, a more extensive resource (e.g., appendix) for the Tool is warranted.

**Discussion:**

A revised Tool was created to improve its comprehensiveness and comprehensibility. Thus, the Tool is a valid resource for applying FES in neurorehabilitation.

## Introduction

Research supports the use of functional electrical stimulation (FES) as an adjunct therapy in neurorehabilitation (1, 2). FES produces sequenced and timed muscle contractions by delivering an electrical current through electrodes on the skin to activate motor neurons, with the purpose of executing functional tasks, such as walking, reaching and standing (1, 3). FES was initially developed in the 1960s as an assistive device (4), such that when stimulation was applied, an instantaneous change in movement or performance would be observed (commonly referred to as an orthotic effect) (5–7). For example, an FES application for foot drop resulted in immediate increases in gait speed in individuals living with the effects of stroke (8). When FES is used to practice a task repeatedly over time, therapeutic gains may be realized, such that improvements in motor performance persist when the electrical stimulation is removed (1, 7, 9–11). For example, FES has been used to achieve lasting improvements in the performance of grasping, reaching, and walking (1, 7–10).

FES is recommended as a therapeutic intervention in best practice guidelines for individuals with stroke and SCI. The Canadian Stroke Best Practice Guideline includes FES as a therapy recommendation to improve both upper and lower extremity function and reduce overall motor impairment in flaccid limbs (9, 12). Likewise, the literature supports the use of FES for individuals with SCI to help address common impairments and activity limitations, such as spasticity, gait impairments, and reduced ability to grasp and reach (10, 11, 13, 14). Despite FES being recommended in several clinical practice guidelines, its use in clinical practice by PTs remains low (3). Auchstaetter et al. (3) assessed how frequently Canadian PTs used FES in stroke rehabilitation and found that among the almost 300 PTs surveyed, most “never” or “rarely” used FES. The PTs reported their lack of knowledge, training, and expertise in FES, and the perceived lengthy set-up time as barriers to FES implementation in clinical practice (3). PTs acknowledged the importance of understanding FES parameters and the opportunity for hands-on training (3). Indeed, being “comfortable and confident in applying FES”(3) was one of the most commonly cited facilitators of FES use amongst PTs who use the modality in their practice.

**Figure 1:**
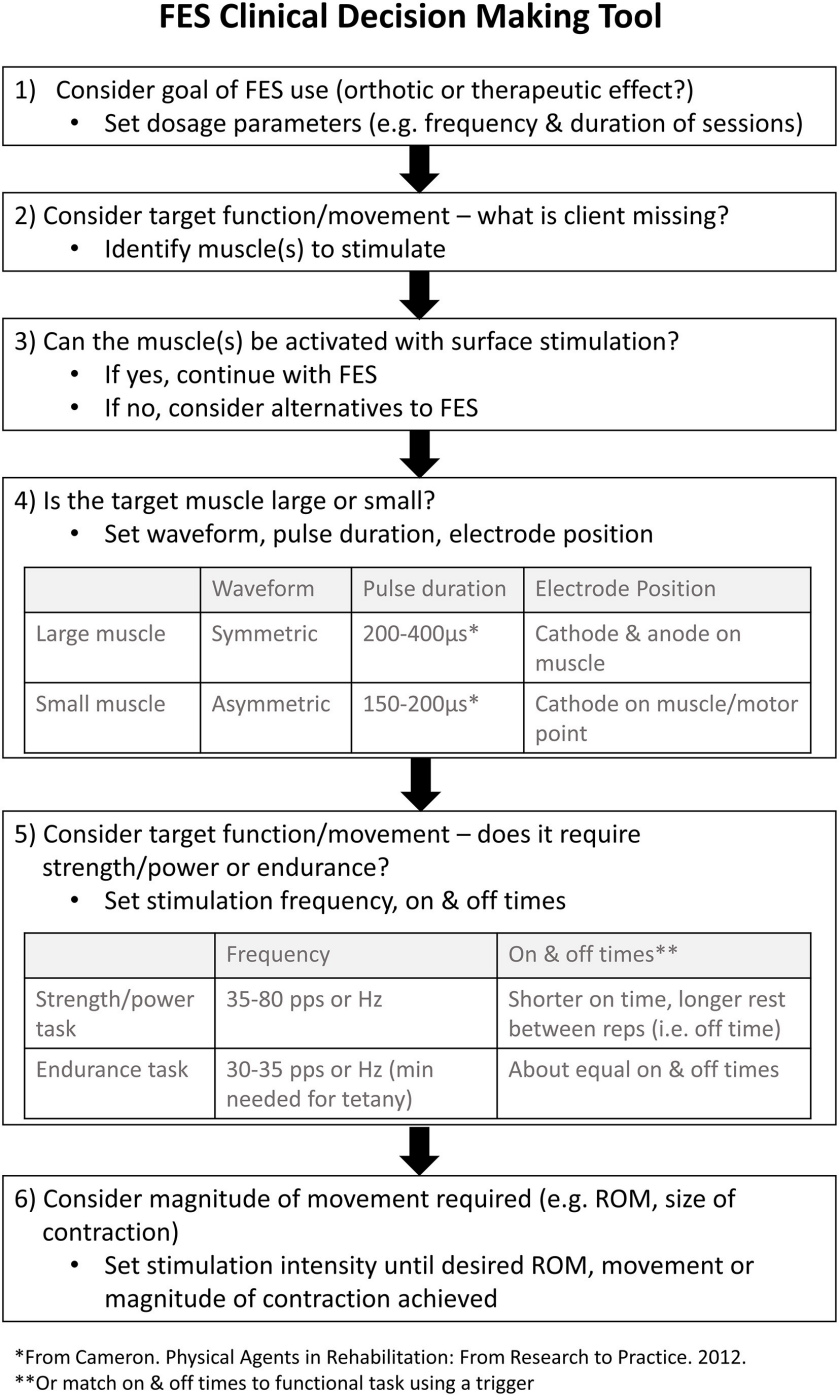
The FES CDM Tool used during semi-structured interviews. The Functional Electrical Stimulation (FES) Clinical Decision Making (CDM) Tool was developed to assist clinicians in the selection of electrical stimulation parameters when applying FES. ROM, range of motion.

To date there is no published guide on how to select electrical stimulation parameters (e.g., frequency, pulse duration/width, waveform, and amplitude) for the myriad of FES applications used with neurological populations. Summaries of the stimulation parameters used in previous research studies have been produced (15), yet there is considerable variety in the prescription of parameters across studies targeting the same orthotic or therapeutic goal. In an effort to contribute to the translation of FES into clinical practice and address the knowledge gap surrounding FES application, a FES Clinical Decision Making Tool (FES CDM Tool) (Figure 1) was developed by a research team member (KEM) through literature review, expert opinion, and clinical experience. More specifically, the electrical stimulation parameters that a clinician may need to set for any given FES application were identified as important to include in a decision-making tool. These parameters included stimulation amplitude, frequency, pulse duration and waveform. To provide recommendations on how to set these parameters, knowledge of the characteristics of electrical currents and their neurophysiological effects were translated into clinically relevant questions to guide a clinician's prescription of FES. This tool aims to help therapists determine the appropriate parameters for a given FES application. However, the content validity of this tool, or the extent to which it reflects the target construct, has yet to be examined. An evaluation of content validity typically considers the appropriateness, comprehensiveness, and comprehensibility of a tool with respect to the construct and population (16). Such an evaluation is a necessary step in the development of clinical decision-making tools, like the FES CDM Tool.

Due to the inclusion of FES in best practice guidelines for neurological populations and the increasing interest in its use in neurorehabilitation (3), creating FES-related resources for clinicians, such as the FES CDM Tool, is a priority. The purpose of this study was to evaluate the content validity of the FES CDM Tool from the perspectives of Canadian PTs and OTs that use FES in neurorehabilitation. Specifically, the study objectives were to (1) understand the factors that PTs and OTs consider when selecting electrical stimulation parameters for applications of FES, and (2) evaluate the content validity of the FES CDM Tool.

## Methods

### Study design

This cross-sectional qualitative descriptive study used individual semi-structured interviews to explore the perspectives of Canadian PTs' and OTs' clinical decision making experiences when applying FES in clinical practice, and evaluated the content validity of the proposed FES CDM Tool. This study received approval from and was conducted in accordance with the established ethical standards of the Health Sciences Research Ethics Board (REB) at the University of Toronto.

### Recruitment

Recruitment notices were circulated through the Neurosciences Division of the Canadian Physiotherapy Association (CPA), the Canadian Association of Occupational Therapists (CAOT), the Canadian Activity-Based Therapy Community of Practice, and KEM's professional network. Prospective participants contacted the research team *via* email and Zoom or telephone interviews were scheduled after informed consent was provided and study eligibility was confirmed.

### Participants

Participants who met the following inclusion criteria were eligible to participate:

1. PTs and OTs who were licensed to practice in Canada.
2. Practiced in the area of neurorehabilitation with >50% of their caseload involving adults or children with upper motor neuron damage.
3. Practiced in any clinical setting (including but not limited to: acute care, rehabilitation hospital, private practice, home care, community care, etc.).
4. Used FES in their clinical practice, at least occasionally (i.e., with a minimum of 21–40% of their neurological clients/patients).
5. Able to participate in an interview conducted in English.

The study aimed to recruit 12–15 participants with at least four participants being OTs. While in Canada the majority of PTs are exposed to FES in the entry-to-practice PT curriculum, most OTs are not and thus, it was anticipated that fewer OTs were using FES in their practice compared to PTs. The target sample size of 12–15 participants aligned with a study by Guest et al. (17), which found that data saturation was largely achieved by the time 12 interviews were analyzed. Despite the research team receiving training for conducting interviews, a lack of previous interviewing experience may have affected the quality of the dialogue, thus a sample size slightly greater than 12 was targeted. Consecutive sampling was used, such that PTs and OTs meeting the eligibility criteria were enrolled in the order in which they self-referred to the study until the target sample size, including at least four OTs, was reached.

### Data collection

Data were collected *via* Zoom or telephone using a demographic questionnaire and semi-structured interview guide. The demographic questionnaire queried participant age, gender, employment status, job title, work setting, province of practice, years of experience as a clinician, years of FES use in practice, and percentage of neurological clients for which FES is used. The guide consisted of 12 open-ended interview questions (Table 1) created by the research team, which included six PT students and a clinician-scientist with FES expertise. The questions were developed based on recommendations of Brod et al. for qualitative research to examine content validity, as well as the interview guide of a similar qualitative study evaluating the content validity of a decision-making tool (16, 18). The first part of the interview queried how participants selected electrical stimulation parameters for FES applications, and explored the factors considered when making clinical decisions. In the second part of the interview, participants were shown the FES CDM Tool (Figure 1). Cognitive debriefing interviewing with a verbal probing approach was used to query the appropriateness, comprehensiveness, and comprehensibility (i.e., content validity) of the FES CDM Tool (16). Participants were asked to explain their interpretation of each step, consider the relevance of each step to setting electrical stimulation parameters, and how easy or difficult it would be to apply each step (16). Interviews were conducted by two team members where one team member interviewed the participant (RF or NA), and the second (CT) reported observations in a reflective journal. Interviews lasted 36–76 min and were audio recorded and transcribed verbatim. Synthesized member checking (SMC) was used to allow participants to provide feedback as to whether the data accurately reflected their experiences (19).

**Table 1:** Semi-structured interview guide.

1.	Please tell me about your clinical practice and your experiences using functional electrical stimulation, or FES, in your practice.
2.	Tell me about the therapy goals that you use FES for.
3.	How did you learn about and gain skills in FES?
4.	We'd like to learn more about how therapists make decisions about setting stimulation parameters, such as frequency, pulse duration and intensity, among others. Can you tell me about your process for setting the stimulation parameters for a client?
5.	One of the researchers on our team, Dr. Kristin Musselman, created a FES Clinical Decision Making Tool that may help clinicians make decisions about how to set electrical stimulation parameters when using FES. I am going to share my screen to show you this tool. There are 6 steps in this tool. I'd like to review each step with you and ask a few questions. The first step is to ‘Consider the goal of FES use'; for example, whether the goal is orthotic or therapeutic. Determining the goal may help the clinician set dosage parameters, such as the frequency and duration of FES sessions. • Do you think clinicians will understand what this step means? Why or why not? • Do you think this is an appropriate step to include? Why or why not?
6.	The second step is to ‘Consider the target function or movement' to determine what the client is missing. This step may help the clinician determine which muscle or muscles to stimulate. • Do you think clinicians will understand what this step means? Why or why not? • Do you think this is an appropriate step to include? Why or why not?
7.	The third step is to ask ‘Can the muscle(s) be activated with surface stimulation?' This step may help the clinician determine whether FES is feasible in a given clinical scenario. • Do you think clinicians will understand what this step means? Why or why not? • Do you think this is an appropriate step to include? Why or why not?
8.	The fourth step is to ask ‘Is the target muscle large or small?' This step may help the clinician set the position of the electrodes, as well as the stimulation waveform and pulse duration. The table outlines how the size of the muscle affects these parameters of electrical stimulation. Please take a moment to review the table for step 4. • Do you think clinicians will understand what this step means? Why or why not? • Do you think this is an appropriate step to include? Why or why not?
9.	The fifth step is to ‘Consider the target function or movement—does it require strength/power or endurance?' This step may help the clinician set the stimulation frequency and on/off times of the stimulation. • Do you think clinicians will understand what this step means? Why or why not? • Do you think this is an appropriate step to include? Why or why not?
10.	The sixth and final step is to ‘Consider the magnitude of movement required'; for example, the range of motion or size of muscle contraction required for the target function. This step may help the clinician set the stimulation intensity? • Do you think clinicians will understand what this step means? Why or why not? • Do you think this is an appropriate step to include? Why or why not?
11.	Do you think the FES Clinical Decision Making Tool captures all considerations when making decisions about how to set stimulation parameters for FES?
12.	Thinking of the FES Clinical Decision Making Tool as a whole, are there any additional comments or suggestions that you would like to share with us?

### Data analysis

Age, number of years as a PT/OT, and number of years using FES in clinical practice were reported as mean (± 1 standard deviation). Nominal demographic data (i.e., work setting, work location, patient populations) were reported as frequency counts. A qualitative conventional content analysis was conducted; a flexible method of analyzing text data where the objective is to describe a phenomenon (20). This method of analysis was deemed appropriate as limited research or theory on FES-related clinical decision making exists in the literature.

The six-step DEPICT model was followed to allow for collaboration of all team members: (1) dynamic reading, (2) codebook development, (3) participatory coding, (4) reviewing and summarizing categories, (5) collaborative analysis, and (6) translating findings (21). First, researchers worked in pairs to read an initial transcript and created marginal notes to highlight relevant sections. A preliminary codebook was developed from the marginal notes and the remaining transcripts were coded. New codes that emerged were discussed and transcripts were re-coded with the updated codebook. Through team discussion, generated codes were arranged into categories and sub-themes based on relatedness, from which overarching themes were explored. Conclusions on the FES CDM Tool's content validity were determined by the synthesized data based on its appropriateness, comprehensibility, and comprehensiveness. These findings were used to inform changes to the existing tool. Rigor was established through reflective journaling, SMC, and an audit trail.

## Results

### Participant demographics

Nine PTs and four OTs (one man, 12 women), aged 40.5 ± 10.3 years, participated. Participants had worked as a PT/OT for 14.9 ± 10.0 years, with seven currently working in Ontario, two in British Columbia, and the remaining four in Saskatchewan, Manitoba, Quebec, and Alberta. Participants had 9.5 ± 9.2 years of experience using FES in practice. Twelve participants worked in rehabilitation hospital settings and one participant worked in a private practice homecare setting. SCI was the most commonly reported patient population in which participants used FES (***n*** = 12), followed by stroke (***n*** = 8), multiple sclerosis (***n*** = 3), brain injury (***n*** = 3), Parkinson's disease (***n*** = 1), and cerebral palsy (***n*** = 1).

Three themes were identified: (1) Clinician Context Influences FES Usage and (2) Parameter Selection in Clinical Practice, which together informed the third theme (3) With Modifications, the FES CDM Tool is a Valid Resource to Inform FES Application. Several sub-themes were identified for each theme. Table 2 provides additional supporting quotes, with Q1, Q2, etc. linking each quote to the relevant text.

**Table 2:** Supporting quotes by theme.

**Sub-theme**	**Quotes**
**Theme 1: Clinician Context Influences FES Usage**	
1a. Experience with FES Use is Variable	Q1: “FES courses at university, they're very short, and are not adequate, at least for very kind of complicated patients.“ (P10) Q2:“[FES course] kind of built my confidence on using more custom parameters and recognizing when something isn't working.” (P07) Q3:“…at [work facility], we are really lucky. We have a lot of support, so we do have a lot of senior therapists that often teach the newer therapists how to use FES.” (P07) Q4:“I did [FES] through school…we had an FES unit where we practiced with partners and such. Um, and then when I got to the clinic, we had COMPLETELY different systems.” (P01) Q5:“Yeah. I mean just the fact that one's an eight-channel device and has, like a lot of protocols built into it already. Compared with a two-channel device that…really limits you in terms of the muscles you're able to activate, as well as the amount of sequences of movements that you're actually able to help your client achieve…I think those are the biggest differences there.” (P12)
1b. Treatment Goals Defined by Clinicians in their Clinical Practice	Q6: “We ordered [FES device for foot drop] for home use. The goal was for therapy, but [patient] did use it as an orthotic sometimes for foot drop.” (P01) Q7:“I would recommend [FES] for people who had decreased strength neurologically.” (P01) Q8:“Toileting is a biggy.” (P10) Q9:“…very focused on walking” (P01) Q10:“…a lot of the time it's around using tools such as utensils, pens. Sometimes it's related to feeding…maybe around using their arm and hand to move or coordinate a gait aid.”(P12)
**Theme 2: Parameter Selection in Clinical Practice**	
2a. Strategies Used for Decision Making	Q11: “Normally I would just look into the research of the particular population…and then I would try it from there…and then just tweak it based off of what the patient was saying so if they're like ‘it's a little bit too intense', maybe this or if they're not getting like a full muscle contraction, maybe upping the duration and such. So just kind of like figuring it out for the particular patient. But I'd always start off with whatever the research was saying.” (P01) Q12:“…looking at their response to FES pain wise” (P07) Q13:“…a spinal cord injured person who has less sensation, you can usually get a little bit more, higher parameters, without them complaining of pain or feeling it.” (P04) Q14:“Tone is sort of dependent on a lot of factors, and if they're stressed, if they're tense, if they're not breathing deeply, where they're looking, neurodynamics…it all plays into how you're gonna activate things.” (P06) Q15:“…basically [looking] at what pattern [was] missing, and what needs to be stimulated to help that person with the functional goal…look at pulse duration, amplitude, ramp, and frequency, you know on and off times based on what the person tolerates, and what gives activation of the specific patterns that you want to do their function.”
2b. Challenges of Selecting Stimulation Parameters	Q16: “I consider scientific recommendations, environmental factors, and person as well…it varies day by day.” (P10) Q17:“So mostly for upper extremity it's very difficult and it's changing so much between people and so you just need to test and try to find the good position, and if it's not working the first time, it's okay. You change it a bit and the response can be completely different.” (P13) Q18:“I've tried it for reaching - I really struggle. I'm still new at this so I'm not great at getting the electrodes in the right place for reaching, but they haven't been overly successful.” (P03) Q19:“I was not as experienced with it. I would sort of like go by the [preset protocols] sort of thing, right? But then, you know, like…after I took the course, I felt steadier about making those decisions.” (P08)
**Theme 3: With Modifications, the FES CDM Tool is a Valid Resource to Inform FES Application**	
3a. Clinician Feedback on the FES CDM Tool	**Step 1** Q20:“Yeah, I think you definitely need [this step] because…all of your settings and everything are going to be based on that.” (P01) Q21:“If you take [KEM's] course…it makes sense. You will know, right? It's just if you didn't take the course, then it might be a little bit, ‘I don't know what to do with this sort of thing'.” (P08) Q22:“I don't know that many people would know how to determine the difference between the two…there could be definitions like explaining what that meant on the tool…that could probably help.”(P12) Q23:“… a good first step. Don't know if this takes into account someone's contradiction and precautions for FES. So typically, in the decision making, I usually make sure that I clear any contraindications and make sure they're aware of the risks and benefits of FES. And that, I think, would be my first step to see if they're even appropriate to even think about using FES.” (P09)
	**Step 2** Q24:“Right, consider the target function movement. What is [the] client missing and yeah, no, I think it's a great step. I think it's very important. If anything, it's the most important step for the clinician…the decision-making, right? It's like, what am I going to stimulate? And allows clinicians, again who are newer to this…don't feel that confident, they may feel like there's too many muscles and ‘no, I don't know where to start'. So, I think that's a great question to help them zero in. Okay, well what are you trying to [do]? You're working on reach. What are they missing for that? Oh, they don't have enough proximal, oh I could do their delts…don't have wrist extension, oh well put it on the wrist extensors right? So it's a, I think it's [a] good question. I would keep it for sure.”(P08) **Step 3** Q25:“The way that is worded, it took a lot of teasing out by [interviewer] to help me understand it. So maybe if it was more just talking about access to the muscle…in terms of superficial, deep. That, I think, would make more sense to me.” (P02) Q26:“…making the wording a little bit more clear on what [the tool] means by “activated” (P07) Q27:“…considering trying to stimulate…those deeper muscles…wouldn't be something that [they] would be even attempting.” (P05) **Step 4** Q28:“I just don't know what a large muscle is and what a small muscle is.” (P12) **Step 5** Q29:“Yeah, I think the use of FES for spasticity is, uh, tricky. Just because my understanding is the research around that…it's variable, and how people respond to that stimuli, you know, whether it actually makes your spasticity worse, or does it fatigue the muscle, [which] reduces the spasticity. But then maybe they can't use it in a functional way or it has other effects, after fatiguing the muscle. So I think it is, it's nice to include it…you know just to again be able to narrow the parameters and make it a lot more specific.” (P07) **Step 6** Q30:“I think it just makes sense in the way the decision tree is laid out as far as you know, if you…choose your muscles, choose your goals, larger, small, looking at that duration and the placement, right? The last thing, naturally, that seems to come is that intensity…how much do you need to get the movement required?” (P07) Q31:“I want to see a contraction. I want to see movement. And I also want to make sure it's comfortable for the person. So I'm not necessarily looking at ‘does this contraction allow them to move through their entire range of motion?' That's something I haven't considered.” (P02) Q32:“I'm thinking, ‘I don't want to hurt them.' I want it to be comfortable. And I definitely want to see it elicit the movement.” (P02)
3b. Addition of an Appendix	Q33: “I agree with adding a little Appendix to define the terms, or like have a bit more narrative around it, maybe. But, not too much, but just like a little bit. Or give an example, maybe, or maybe a picture or two.” (P08) Q34:“I like I like how simple the tool is and I agree that it's probably best to not make it too cluttered…so having an Appendix if someone needs to refer to a little bit more…would be great.” (P09) Q35:“I think [for an Appendix] having photos of the different movements is obviously a huge added bonus, um contraindications, or like, who is appropriate…a preface saying what FES is, and maybe some of the research behind it…especially how often should you be using it?…Could also talk about the different types of machines too, symmetric vs. asymmetric…You could also do some case studies…You know just little things like that to make people think. Again, I guess it depends how comprehensive you want the tool to be, but when people are starting to use this tool, these are all things that they need to think about right?” (P07) Q36:“A lot of people are worried about contraindications and so…to have them all laid out there on it, like right in this document would be really helpful as a quick reference just to make them more comfortable to use it.” (P03)

### Theme 1: Clinician Context Influences FES Usage

Participants described their FES usage as dependent on their clinical context. The influences were grouped into two sub-themes. Participants explained their (a) Experience with FES Use is Variable, and dependent on their individual learning strategies and the devices they used. The (b) Treatment Goals Defined by Clinicians in their Clinical Practice included both therapeutic and orthotic goals and guided FES use.

#### Sub-theme 1a: Experience with FES Use is Variable

Across participants, experience with FES varied. Participants explained that FES use was influenced by access to FES education and devices. With respect to education, participants reported that Canadian entry-to-practice programs in PT and OT either did not include or did not adequately teach FES applications (Q1). Thereby, many participants reported using alternative learning methods such as reading literature to help inform parameter selection and appropriate clinical populations (*n* = 4). Additionally, many participants reported learning through post-graduate FES training (*n* = 9) (Q2), their clinical experience (*n* = 8), patient feedback (*n* = 8), and mentorship from colleagues (*n* = 3) (Q3).

Participants reported using different FES devices that varied in the number of channels and the ability to modulate each parameter. The device one was familiar with affected clinical practice (Q4). The differences in the devices can impact how FES is used. Depending on the device, the movements that can be practiced and the level of input from the PT/OT (e.g., parameter selection) can change (Q5). For example, a FES device with preset protocols requires minimal parameter selection.

#### Sub-theme 1b: Treatment Goals Defined by Clinicians in their Clinical Practice

Participants described using FES to address various treatment goals in their clinical practice, impacting FES usage. Participants primarily reported using FES for therapeutic rather than orthotic treatment goals. Eight participants reported using FES for orthotic use, at least occasionally, with the primary goal being to correct foot drop (*n* = 7) (Q6). Seven participants reported using FES to address spasticity. Therapeutic goals also included strength and endurance training (Q7) and functional goals (Q8–10).

### Theme 2: Parameter Selection in Clinical Practice

Participants described their clinical decision making when selecting electrical stimulation parameters for FES applications. The Parameter Selection in Clinical Practice theme included two sub-themes: (a) Strategies Used for Decision Making (i.e., literature, patient feedback, matching to functional goals, using the same parameters, following a tool), and (b) Challenges of Selecting Stimulation Parameters.

#### Sub-theme 2a: Strategies Used for Decision Making

Participants described their clinical decision making strategies for setting up an FES application; this included referring to the literature and incorporating patient feedback to adjust parameters (Q11), including input on pain (Q12) and tolerance of the stimulation. Further, a patient's level of sensation affected parameter selection (Q13). Additionally, participants described considering patients' biopsychosocial factors (Q14) and functional goals (Q15).

Approaches for parameter setting ranged from structured approaches including preset parameters to unstructured approaches, such as using “trial-and-error” (P06 and P07). Some participants did not alter parameters at all either due to following literature guidelines, limitations of the FES device setting options, or they habitually used the same parameters (P01, P03, P09, P10, and P13). Other participants described a process where they would have guidelines in mind, either from the literature or frameworks that they learned about in previous courses, and then altered parameters based on their understanding of each parameter and patient comfort (P02, P04, P05, P08, P11, and P12). Lastly, P06 and P07 described a process for setting up FES parameters that was rooted in principles, but involved alterations and trial-and-error to get the desired outcome.

#### Sub-theme 2b: Challenges of Selecting Stimulation Parameters

Participants discussed the decision making challenges previously experienced when setting up FES, such as how “finicky” parameter setting was (P11 and P13) and the variability across and within patients day to day (Q16–17). Interestingly, the participants' level of confidence in FES application influenced their clinical decision making (Q18–19).

### Theme 3: With Modifications, the FES CDM Tool is a Valid Resource to Inform FES Application

Overall, the participants reported the FES CDM Tool to be valid, but they suggested modifications to improve its clarity. Two sub-themes were identified within this theme. First, (a) Clinician Feedback on the FES CDM Tool included the strengths, limitations, and suggested additions and changes for each step of the tool. Second, most participants recommended the (b) Addition of an Appendix that would contain more FES resources for clinicians without cluttering the simplicity of the tool itself.

#### Sub-theme 3a: Clinician Feedback on the FES CDM Tool

##### Step 1

Step one of the FES CDM Tool asks clinicians to determine their goal when using FES (i.e., orthotic or therapeutic) in order to help set dosage parameters (i.e., frequency and duration of sessions) (Figure 1). Participants described step one of the FES CDM Tool as understandable and an appropriate step to include (*n* = 12) (Q20). In contrast, P06 stated they did not find step one to be understandable due to the limited use of FES as an orthosis in Canada. Participants reported that one limitation of this step is whether clinicians will understand the difference between therapeutic and orthotic goals (*n* = 4) (Q21–22). Three participants suggested adding a section on contraindications or patient appropriateness, specifically, within or prior to step one (Q23).

##### Step 2

Step two prompts the clinician to consider the target function or movement to then determine what muscle(s) they need to target with the FES (Figure 1). Twelve participants noted that this step was both understandable and appropriate (Q24).

In contrast, P10 noted that identifying the muscles to stimulate was appropriate, however, the prompt ‘what is the client missing?' was “extra, just not helpful.” Another suggested change to step two was to modify the wording to include a spasticity-related goal, since spasticity is not what a patient is missing but rather something that a patient has too much of (P13).

##### Step 3

Step three asks the clinician to consider if the muscle(s) being targeted can be activated with surface-level stimulation (Figure 1). While participants understood this step and felt that it was appropriate (*n* = 11), some required further prompting from the interviewer to help interpret it (Q25–26). For example, three participants highlighted how this step could be interpreted to be asking if FES is appropriate given the patient's diagnosis. P12 suggested that adding in examples may help clarify the step's intended purpose and meaning. In contrast, two participants did not think this was a necessary step (Q27).

##### Step 4

Step four asks the clinician to consider whether the target muscle is large or small in order to set the waveform, pulse duration, and electrode position (Figure 1). Many participants described that they understood this step and it was an appropriate step to include (*n* = 12). In contrast, P06 stated the table provided in this step is “not as simple as what it's listed as…because it's person specific to some degree.” Additionally, P12 suggested adding examples either into the step or an Appendix of how to differentiate muscle size for setting parameters (Q28).

##### Step 5

The fifth step is to consider if the target function or movement requires strength/power or endurance to set the stimulation frequency and on/off times (Figure 1). Many participants found this step understandable and appropriate (*n* = 12). Similar to step four, P06 stated they often selected frequencies outside of the given ranges recommended in step five depending on the patient's response to the simulation. The most common feedback received for this step was to add guidance around using FES for spasticity management (*n* = 6). However, some participants were not convinced spasticity management should be included in the FES CDM Tool (Q29).

##### Step 6

The final step of the FES CDM Tool is to consider the desired magnitude of movement in order to adjust the current intensity to achieve the desired range of motion, movement, or magnitude of muscle contraction (Figure 1). Ten participants understood the step and confirmed its appropriateness (Q30). One participant misinterpreted this step to suggest the stimulation should achieve full range of motion (Q31). Five participants noted the importance of taking into account patient factors when increasing the intensity such as skin sensation, fatigue, psychosocial considerations, and comfort (Q32). Four participants wanted the step to state that the patient should be actively performing the movement in conjunction with the muscle stimulation.

#### Sub-theme 3b: Addition of an Appendix

During the interviews, all participants expressed a need for FES resources in the form of an Appendix to the FES CDM Tool (*n* = 13) (Q33–35). The most frequent suggestion was to include more information about pad placement (*n* = 6). Another suggestion was to include contraindications to FES (*n* = 5) (Q36). Four participants suggested clarifying small vs. large muscles. Some less common suggestions were to include more information about: pad size (*n* = 2), commonly targeted muscles (*n* = 2), frequency and duration of sessions (*n* = 2), parameters for spasticity (*n* = 2), photos depicting different ways to use FES (*n* = 2), various FES devices (*n* = 2), case studies (*n* = 2), and definitions of common terms such as orthotic and therapeutic goals (*n* = 3).

## Discussion

This study evaluated the content validity of the FES CDM Tool from the perspectives of Canadian PTs and OTs using FES in neurorehabilitation. Participants described previous academic and clinical experiences that influenced their use of FES. Although they had varying experiences, participants reported the FES CDM Tool to be appropriate and suggested modifications to increase its comprehensiveness and comprehensibility. Participants emphasized the importance of a FES CDM Tool being accessible for clinicians working in neurorehabilitation and encouraged future development of resources to support its use in clinical practice.

Clinical decision making when applying FES appeared to be challenging for study participants due to the lack of FES education and resources. The strategies used to select and manipulate parameters varied, with most participants using preset programs or trial-and-error when applying FES. This may have resulted from a lack of confidence in altering parameters and/or device selection. Four participants utilized devices with preset parameters, meaning they had little opportunity to customize the parameters for each patient. However, few participants articulated an evidence- or knowledge-informed approach to parameter selection; for example, participants did not discuss the relationship between stimulation frequency and muscle fatigue or the effect of increasing pulse duration/width on motor unit recruitment.

The FES content provided in Canadian PT and OT entry-to-practice programs was perceived as insufficient to prepare clinicians to apply FES. Similarly, Australian PTs and OTs reported gaining skills in FES application through post-graduate education and mentoring rather than entry-level education (22). A lack of knowledge, training, and expertise in FES is known to be the main barriers PTs and OTs face when applying FES (3, 23). In the present study, some participants attributed their increased confidence in parameter setting and manipulation to attending post-graduate courses. This aligns with prior research suggesting that one of the main facilitators to enabling FES use in clinical practice are increases in access to continuing education and the implementation of programs to increase awareness of FES application (23).

**Figure 2:**
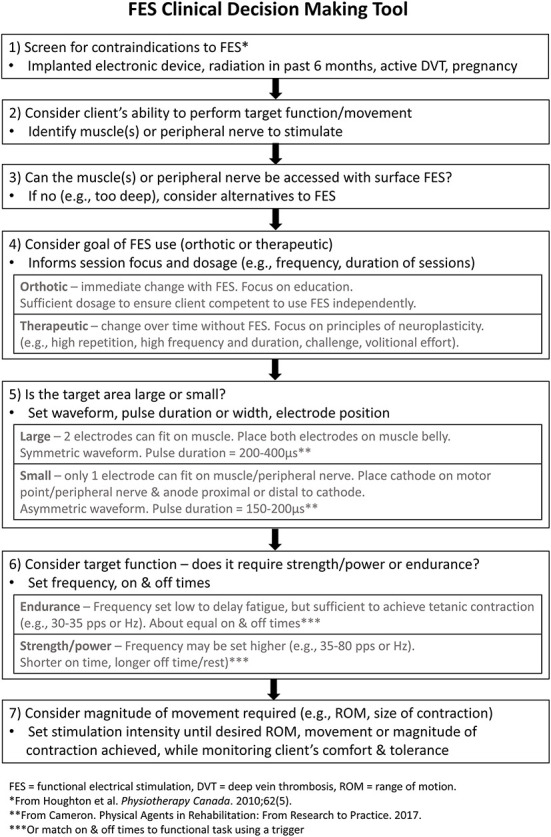
The revised FES CDM Tool. The revised Functional Electrical Stimulation (FES) Clinical Decisions Making (CDM) Tool, which incorporates the revisions and additions suggested by study participants.

The findings from this study identified several suggestions to improve the FES CDM Tool that have been implemented to create a revised version (Figure 2). Wording was revised to increase clarity; for example, asking clinicians to consider whether the target muscle or peripheral nerve can be accessed, rather than activated, by surface-level stimulation, as step 3 in the original FES CDM Tool proved difficult for some participants to understand (Figure 1). An explanation of small vs. large target areas for the stimulation was added to step five (Figure 2), along with a statement about the placement of the anode electrode when using an asymmetric waveform. For step seven (Figure 2), text was added to remind clinicians to consider the patient's comfort and tolerance as the stimulation intensity is increased.

In addition to editing the FES CDM Tool's wording, some conceptual changes were made. First, a step was added to prompt screening for potential contraindications to electrical stimulation [(step 1), Figure 2] (24, 25). This step is followed by identifying the target muscle(s) or peripheral nerve [(step 2), Figure 2] and determining whether the target muscle(s) or nerve can be accessed with surface-level stimulation [(step 3), Figure 2]. This order reflects the logical order of considerations when determining whether a specific FES application is safe and appropriate for an individual living with neurological injury or disease.

Second, definitions of therapeutic and orthotic goals were added to increase clarity of these terms [(step 4), Figure 2], since they are commonly used in FES literature and practice (26). When FES is being used as an orthotic assist, an assessment is typically completed to determine the optimal parameter settings for the FES application (27). In contrast, when FES is being used to achieve a therapeutic effect, electrical stimulation is incorporated into motor training that is characterized by principles of neuroplasticity, such as a high number of movement repetitions, sufficient motor challenge, volitional effort, and frequent practice (28). Hence, several dosage parameters, such as the frequency and duration of sessions, are dependent on the FES goal selected. Defining the terms orthotic and therapeutic may allow for increased understanding of this step, especially by clinicians not familiar with the FES literature. Interestingly, participants of the present study described using FES for primarily therapeutic goals. While using FES as an orthotic assist may not be common in Canada, it is frequently used for this purpose in other geographical regions, such as the United Kingdom (29). One possible reason for these regional differences is the availability of funding for FES devices. In Canada, both clinicians (3) and recipients of FES (30) have reported a lack of funding for patients to acquire FES devices, another barrier to using FES.

Third, when discussing the final step in the original FES CDM Tool, four participants highlighted the importance of the patient voluntarily moving along with the electrical stimulation. This is an important concept to convey in a FES CDM Tool as FES combined with voluntary movement results in greater brain activation than FES alone (31, 32). Hence, encouraging patients to actively participate in the movement while receiving electrical stimulation may promote neuroplasticity and therapeutic goals. The importance of volitional effort is now mentioned in step four in the revised FES CDM Tool (Figure 2). These revisions were made to improve the Tool's comprehensiveness and comprehensibility, both of which contribute to a tool's content validity (16). We expect the FES CDM Tool may require additional modification in the future in response to new knowledge, new technology or changing practice patterns. Hence, the FES CDM Tool should be viewed as a living document that will be re-evaluated and revised as needed.

Participants suggested including FES applications for spasticity in the FES CDM Tool. Both sensory-level (i.e., transcutaneous electrical nerve stimulation (TENS)) and motor-level electrical stimulation (i.e., FES, neuromuscular electrical stimulation (NMES)) may be used to reduce spasticity in neurological populations. Current clinical practice guidelines for stroke rehabilitation indicates mixed evidence for the effects of NMES and FES on spasticity (9). In contrast, the guidelines indicate that TENS significantly reduces spasticity (9). Hence, if the primary goal of using electrical stimulation in post-stroke rehabilitation is to reduce spasticity, TENS would likely be more effective than FES. In SCI rehabilitation, however, TENS and FES have been shown to have similar anti-spasticity effects after a single session of electrical stimulation (33). Since FES is not more effective than TENS for spasticity management, we have not edited the FES CDM Tool to include a focus on spasticity. Moreover, the steps of the FES CDM Tool could be followed to set up an FES application to reduce spasticity; the evidence-based principles of motor-level electrical stimulation reflected in the Tool still apply.

All participants identified a need for additional resources pertaining to FES applications. While participants identified the FES CDM Tool as a useful resource to help guide clinicians in their clinical reasoning for FES application, they indicated that a more extensive resource, such as an Appendix for the FES CDM Tool, is warranted. These findings are consistent with the findings of Auchstaetter et al. (3), who reported a lack of readily available resources for FES as a barrier for FES application in stroke rehabilitation. Improving access to resources may promote confidence with FES application and facilitate its implementation into clinical practice. While the FES CDM Tool and an accompanying Appendix may support implementation, these resources are not expected to replace the need for formal education (i.e., FES education in entry-to-practice programs, continuing education courses) or informal training (e.g., mentoring by colleagues).

While a lack of educational resources and training on FES is a commonly reported barrier to FES use (3, 23), there are numerous additional barriers to consider. For example, a lack of time with patients, the perceived lengthy set-up time of FES, therapist preference for other treatment options and cost for the user are additional barriers that have been reported by health care providers working in stroke (3) and SCI (23) rehabilitation. According to the Knowledge-to-Action Framework, clinicians interested in using FES in their clinical practice should identify barriers in their local clinical context and then select the implementation strategies best suited to address these barriers (34). On its own, an educational resource like the FES CDM Tool will not address the majority of barriers to FES implementation. The Tool may provide clinicians with knowledge and greater confidence in applying FES, as well as reduce the time spent on setting up and changing FES parameters; however, these potential effects will need to be evaluated in future research.

This study had potential for bias as Canada consists of a small community of clinicians who utilize FES within their clinical practice. Some participants were already familiar with the FES CDM Tool since they took KEM's post-graduate FES courses. To mitigate this potential bias, KEM was not involved in the data collection and analysis for this study.

In conclusion, Canadian PTs and OTs using FES in their clinical practice indicated that the FES CDM Tool is appropriate in guiding clinical decision making for FES applications, supporting the tool's content validity. A revised version of the FES CDM Tool was created to improve the tool's comprehensiveness and comprehensibility. The FES CDM Tool may help clinicians working with neurological populations to confidently apply FES during neurorehabilitation sessions in a safe, effective, and patient-centered manner.

## Data availability statement

The datasets presented in this article are not readily available because of ethical and privacy restrictions. Requests to access the datasets should be directed to Daniel Gyewu (Research Ethics Manager, Health Sciences), d.gyewu@utoronto.ca.

## Ethics statement

The studies involving human participants were reviewed and approved by the Health Sciences Research Ethics Board (REB) at the University of Toronto. The patients/participants provided their written informed consent to participate in this study.

## Author contributions

NA, SC, and RF contributed to design of study, data collection and analysis, and wrote the first draft of the manuscript. CT, KC, and SD contributed to design of study, data collection and analysis, and manuscript revisions. KM contributed to conception and design of study and manuscript revisions. All authors approved the submitted version.

## Funding

Funding for this study was provided through the Canada Research Chairs Program (CRC-2020-00193) and the Coriat Family Research Fund to KM.

## Conflict of interest

The authors declare that the research was conducted in the absence of any commercial or financial relationships that could be construed as a potential conflict of interest.

## Publisher's note

All claims expressed in this article are solely those of the authors and do not necessarily represent those of their affiliated organizations, or those of the publisher, the editors and the reviewers. Any product that may be evaluated in this article, or claim that may be made by its manufacturer, is not guaranteed or endorsed by the publisher.
